# Somatic mutations and genome mosaicism in aging and disease

**DOI:** 10.1038/s12276-026-01791-3

**Published:** 2026-08-07

**Authors:** Elizabeth Pan, Alexander Maslov, Jan Vijg

**Affiliations:** Albert Einstein College of Medicine, Bronx, NY USA

**Keywords:** Genomic instability, Mutation

## Abstract

Age-related genome mosaicism is an inherent feature of multicellularity and genomic instability. It occurs because of DNA mutations, the accumulation of which leads to diverse genomic landscapes across different tissues. DNA mutations in the genome are consequences of DNA damage, changes in the chemical structure of DNA, such as strand breaks or loss of bases. DNA damage is very frequent and normally repaired quickly. However, errors intrinsic to DNA repair or replication can give rise to permanent changes in genome sequence information. Such DNA mutations are diverse and include single-nucleotide variants, small insertions and deletions, and larger genome structural variants. Since the 1950s, somatic mutations have been proposed to be a major cause of aging. Indeed, somatic mutations are the cause of cancer, the risk of which increases exponentially with age, and possibly other age-related diseases, such as neurodegenerative diseases and cardiomyopathies. Somatic mutations vary from cell to cell owing to the innate stochasticity of their occurrence, from error-prone processing of randomly inflicted DNA damage. With the emergence of single-cell and single-molecule sequencing, it has become possible to quantitatively analyze somatic mutations in human cells and tissues. Here, we discuss a possible causal relationship between mutation-driven mosaicism of the somatic genome and aging-related functional decline and disease by exploring several predictions of the somatic mutation theory of aging.

## Age-related genome mosaicism drives functional decline and disease

The inherent instability of genome increases with age, leading to the accumulation of mutations and resulting in genome mosaicism, in which genetic diversity exists within an individual’s cells. This Review explores the role of somatic mutations in aging and age-related diseases, examining their origins and potential impacts. The authors discuss methods for studying mutations, including next-generation sequencing, and highlight findings from studies on human progeroid syndromes and long-lived species. Results indicate that somatic mutations accumulate with age, contributing to functional decline and increased disease risk. For example, somatic mutations in liver cells rise significantly with age, potentially due to the detoxification role of the liver. These findings suggest that understanding somatic mutations could provide insights into aging mechanisms and inform future research directions, such as improving DNA repair to mitigate mutation accumulation and its effects.

This summary was initially drafted using artificial intelligence, then revised and fact-checked by the author.

## Introduction

The genome, serving as the primary blueprint for cellular function, exhibits a progressive propensity toward instability as an organism ages. This temporal deterioration is characterized by the gradual accumulation of irreversible genome sequence alterations, termed mutations. The cumulative effect of mutations gives rise to the emergence of genome mosaicism, a phenomenon wherein the genetic composition of cells within a single individual becomes increasingly heterogeneous. This concept is an intrinsic feature of the complexity of multicellular life, in which diverse genetic landscapes coexist within a single organism across lineages of cells.

In the early 1900s, before the elucidation of the role of DNA in cellular processes, Dutch botanist Hugo de Vries introduced the term mutation to explain the sudden organismal variations in evening primrose^1^. Two decades later, the earliest concepts of genome mosaicism began to emerge when Boveri^2^, Morgan et al.^3^, and others suspected that cancer was caused by genetic mutations. During this period, scientific consensus began to arise that mutations could alter the composition and functionality of genes, the fundamental units of heredity^4^. Forty years later, based on observations of radiation-induced carcinogenesis and accelerated aging in mammals, Failla^5^ and Szilard^6^ independently postulated that the accumulation of de novo somatic mutations could logically explain the underlying etiology of both aging and cancer. However, their predictions were only based on applied mathematical models using mortality data, as direct measurement of mutation was not feasible at that time.

In the immediate aftermath, cytogenetic studies using Giemsa staining of metaphase chromosomes revealed a positive correlation between organismal age and the frequency of chromosomal aberrations in human lymphoid cells, linking genome mosaicism to aging^7^. In 1976, Nowell^8^ was able to illustrate the process of metastatic tumor formation, demonstrating how cancer can occur through iterative cycles of mutation acquisition and clonal selection of mutated cells. Subsequent to those initial findings, research incrementally confirmed the pivotal role of somatic mutations in oncogenesis and their potential implication in diverse age-associated pathologies. We now know that somatic mutations are both irreversible and unavoidable. Although these findings solidified the role of post-zygotic mutations in carcinogenesis, a causal role of somatic mutations in the gradual decline of physiological function and increased disease risk during aging remained a subject of ongoing investigation.

This Review briefly examines the mechanistic origins of mutations and their potential role in aging and age-related diseases. We explore predictions of the somatic mutation theory of aging and methods for studying and quantifying mutations in vivo. We discuss possible mechanisms through which somatic mutations and somatic mosaicism can have causal roles in the functional decline and increased disease incidence with age. Our discussion covers current understanding of germline and somatic mutation, incorporating insights from next-generation sequencing (NGS) technologies. We also consider how research on human progeroid syndromes, long-lived human subjects, and animal species with extreme longevity, such as bowhead whales, may provide insight into the molecular basis of aging.

## Mutations: molecular end points of DNA damage and faulty genome maintenance

**Fig. 1:**
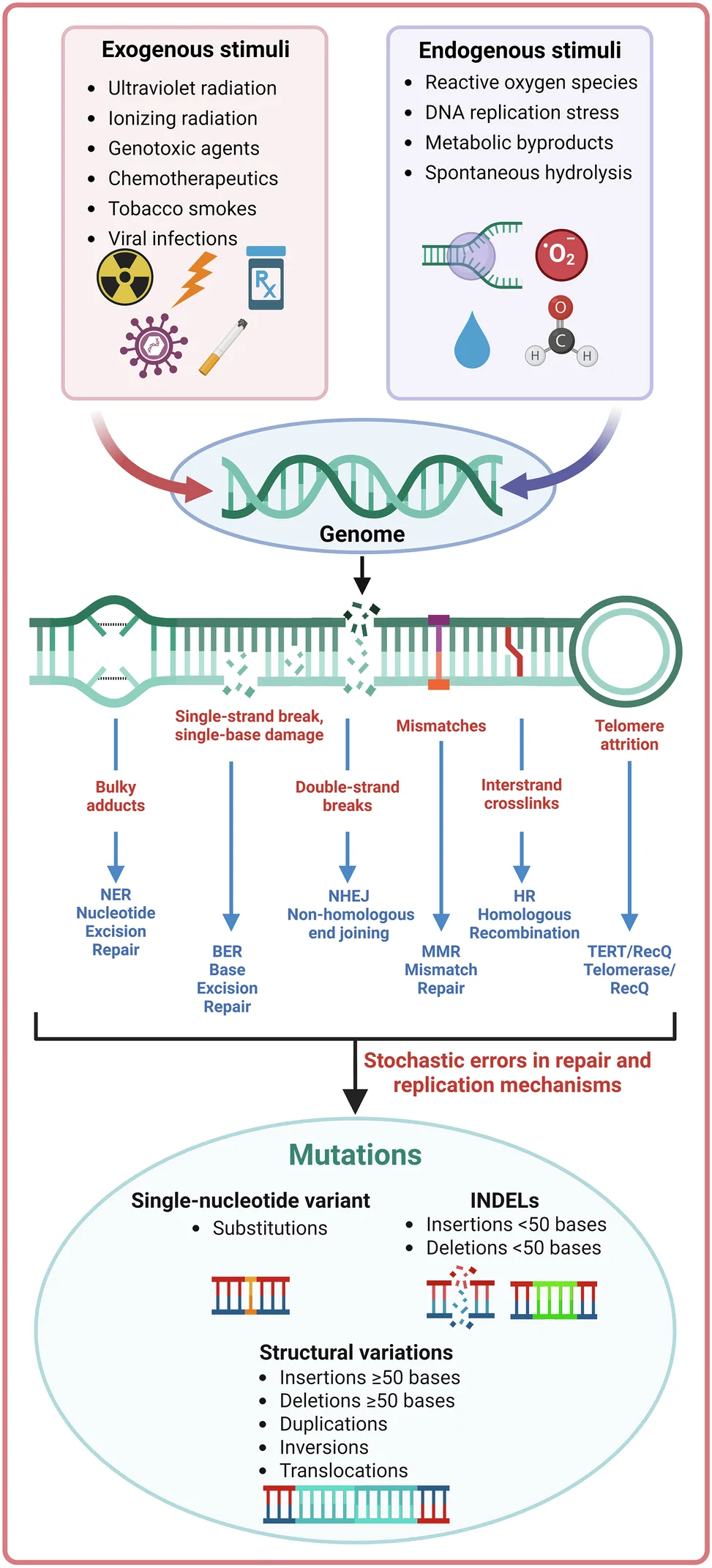
Mutations are the final end points of genotoxic stimuli. Various exogenous and endogenous factors ubiquitously present in our environment or caused by cellular processes can induce a wide range of DNA lesions that compromise the integrity of the genome. Specialized DNA repair mechanisms are rapidly activated to maintain and safeguard the genome from this perpetual assault. Despite this, the inherent imperfections in DNA replication and repair mechanisms inevitably give rise to de novo mutations, presenting irreversible alteration of the genome sequence. These mutations range from single-nucleotide variants, such as base substitution, to more extensive structural variants, such as chromosomal aberrations. Figure created using BioRender.

All mutations, whether in germ cells or somatic cells, are molecular end points of genotoxic stimuli, which can broadly be categorized as endogenous or exogenous. Under physiological conditions, cellular compartments are mostly aqueous and oxygen-rich, with relatively neutral pH (7.4). Under these conditions, the molecular makeup of deoxyribonucleic acids is intrinsically labile, thus vulnerable to various types of DNA damage from both endogenous and exogenous sources^9^. Although exogenous genotoxic stressors are notably potent, endogenous insults from the DNA’s immediate surroundings are persistent and unrelenting (Fig. 1). In other words, under normal conditions, endogenous sources of DNA damage are much more destructive than exogenous elements. Indeed, hydrolytic deamination of DNA bases and cleavage of the glycosidic bond between DNA base and sugar-phosphate group are the most prevalent DNA lesions^10^. In mammalian cells, it has been estimated that roughly 10,000 depurinations and 500 depyrimidinations occur per cell per day. Furthermore, common cellular metabolites such as nitric oxide, formaldehyde, and hydrogen peroxide regularly cause nitrosylation, alkylation, and oxidative damage to our genome^10^.

Although internal cellular processes can compromise genome integrity, external agents can also pose significant threats. Pyrimidine dimers, induced by ultraviolet (UV) light, along with other photoproducts, are the most prevalent DNA lesions observed in environmental context. These lesions predominantly occur in the human skin when UV radiation from the sun induces covalent bonding between adjacent pyrimidine bases, leading to bulky distorted DNA helices^11^. If not efficiently repaired, these structural aberrations can obstruct normal DNA replication and transcription. Beyond the direct effects of damage to the DNA, UV radiation, along with many exogenous agents, can also induce indirect damaging effects. These additional insults transpire via excitation of cytosolic constituents, thus generating reactive oxygen species and reactive nitrogen species, causing genome oxidative stress^12^. In a similar manner, though mainly affecting pulmonary tissue, tobacco smoke induces a wide spectrum of DNA damage owing to its complex array of carcinogenic and mutagenic compounds. Despite its infrequent occurrence, ionizing radiation (IR) can induce highly potent single-strand breaks and double-strand breaks in the DNA phosphate backbone^13^. These breaks often instigate significant shifts and displacement of larger DNA fragments, resulting in cellular detriments and dysfunctions. There are various sources of IR in our daily lives, including medical diagnostic and treatment procedures such as X-rays and chemotherapeutic agents, as well as cosmic background radiation. The ubiquity and potency of both endogenous and exogenous agents underscore the constant assault on cellular DNA. Fortunately, eukaryotic cells have evolved highly robust repair and maintenance mechanisms to counteract the persistent threat of genome instability.

A diverse array of specialized genome maintenance mechanisms are activated and tailored for repairing various types of DNA lesions, averting genome instability and its potential detriments^14^. For instance, chemical alteration of DNA bases caused by oxidation or alkylation is corrected by base excision repair mechanisms^15^, whereas bulky adducts caused by UV radiation can be excised by nucleotide excision repair^16^. Base–base mismatches and insertion/deletion mispairs, generated during DNA replication and recombination, are corrected by mismatch repair mechanism^17^. Interstrand crosslinks and IR-induced double-strand breaks are resolved by either homologous recombination^18^ or non-homologous end joining^19^, depending on the cell-cycle phase and the availability of sister chromatids. Finally, the gradual shortening of telomeres owing to repeated cycles of DNA replication can be prevented or mended by telomerase (Fig. 1). These repair mechanisms may operate simultaneously and compensate for the deficiencies of one another. However, although robust, they are not infallible. Occasional errors can occur, leading to permanent alteration of the genome sequence information. Such events are called mutations.

When occurring in the germline, mutations become the substrate of evolution, which is why mutations have been critical factors in generating the great diversity of life forms on Earth. Although most mutations have either no effect or adverse effects, germ line mutations can also cause genetic disease, such as cystic fibrosis, Huntington’s disease, Down syndrome (DS), or Duchenne muscular dystrophy. There are more than 6000 human genetic diseases^20^.

## Why somatic mutations are difficult to study and ways to detect them

As discussed earlier, mutations are irreversible changes in the sequence organization of the genome. This contrasts with the previously discussed potentially repairable DNA damage. Mutations arise stochastically, reflecting the random nature of DNA damage events and the inherent infidelity in DNA replication and repair mechanisms. When mutations occur in germ cells, they can end up in all somatic cells. With our current advanced sequencing systems, we can easily sequence the whole genome of parents and offspring and find all de novo mutations in a child. By taking that approach, it has been found that each newborn has acquired about 60 new mutations in its genome, mostly derived from the sperm of the father^21^.

However, as mentioned, mutation also occurs in somatic cells, post-zygotically, in a random manner. Consequently, over the course of the aging process, each cell within a multicellular organism acquires a unique mutational profile, which is shaped by its specific cellular environment, exposure to mutagenic stimuli, and the efficacy of the DNA damage response. This phenomenon inevitably results in genomic mosaicism within the soma, in which distinct cell populations and tissues harbor diverse mutational landscapes. Historically, the stochastic nature of this process has posed a substantial challenge to study somatic mutations, except in clonally expanded lineages, such as tumors^22^.

The only types of mutations that could be studied since the 1960s are large chromosomal aberrations. Indeed, early cytogenetic research made significant strides in visualizing chromosomal abnormalities in tumor and normal tissues at the single-cell level. The introduction of Giemsa staining in the 1960s revolutionized the field by producing distinct banding patterns (G-bands) for each of the 24 human chromosomes (22 pairs plus 2 different sex chromosomes). Though with limited resolution, this technique allowed the identification of specific chromosomal regions and the detection of alterations, such as translocations, duplications, and deletions. It was at that time when the first papers appeared reporting an increase with age of chromosomal abnormalities in blood lymphocytes and bone marrow cells^23,24^. This also provided some of the earliest insights into the genetic basis of cancer (chronic myeloid leukemia) and hereditary disorders (trisomy 21 in DS). Over time, the more accurate and versatile fluorescence in situ hybridization (FISH) method would be favored as it enables higher resolution and even the detection of alterations outside of metaphase^25^.

In subsequent decades, reporter systems were developed to detect smaller types of mutations, most prominent being the X-linked HPRT (hypoxanthine-guanine phosphoribosyltransferase) gene assay, which selects for HPRT inactivation by 6-thioguanine resistance. In this system, the numbers of cells carrying mutated HPRT serve as a general representation of overall genome instability. However, the assay is time-consuming and can only be applied to cell types that can be cultured and cloned. Only with the emergence of transgenic reporter genes in mice did it become possible to easily analyze tissue-specific mutation frequencies and spectra during aging or in response to treatment with mutagens^26–28^. These systems have now become the industry standard in genetic toxicology testing^29^.

The disadvantages of somatic mutation analysis at reporter loci are that no direct sequence information is obtained about the genome and that human studies remain beyond reach. With the emergence of NGS, this situation changed. It now became possible to sequence clones, derived from single cells, such as tumors. This resulted in The Cancer Genome Atlas (TCGA), a systematic effort to sequence human tumors for a broad range of mutation types. Thus far the project has sequenced well over 20,000 primary cancers and matched normal samples from 33 cancer types (https://www.cancer.gov/ccg/research/genome-sequencing/tcga). Later, clones from normal cells expanded in culture were sequenced for somatic mutations^30,31^. Each of these clones inherits all the mutations present in the corresponding original cells. Thus, they can serve as effective surrogates for single-cell analysis^32^. However, the process of growing clones is labor-intensive and mostly confined to stem cells.

Single-cell whole-genome sequencing emerged in 2012 as a technique for detecting mutations. For example, Gundry et al.^33^ and Zong et al.^34^ used single-cell multiple-displacement amplification or multiple annealing and looping-based amplification cycles, respectively, for whole-genome amplification of individual cells, followed by whole-genome sequencing. Since then, refinements have been made, most notably primary template-directed amplification^35^, and the usage of error correction software, that allow accurate single-nucleotide variant (SNV) mutation calling^36,37^. Although single-cell sequencing approaches for mutation detection have now been optimized and are widely applied, they are not without limitations. Single-cell sequencing is hampered by its high cost and heightened vulnerability to technical biases, owing to minimal sample input.

**Fig. 2:**
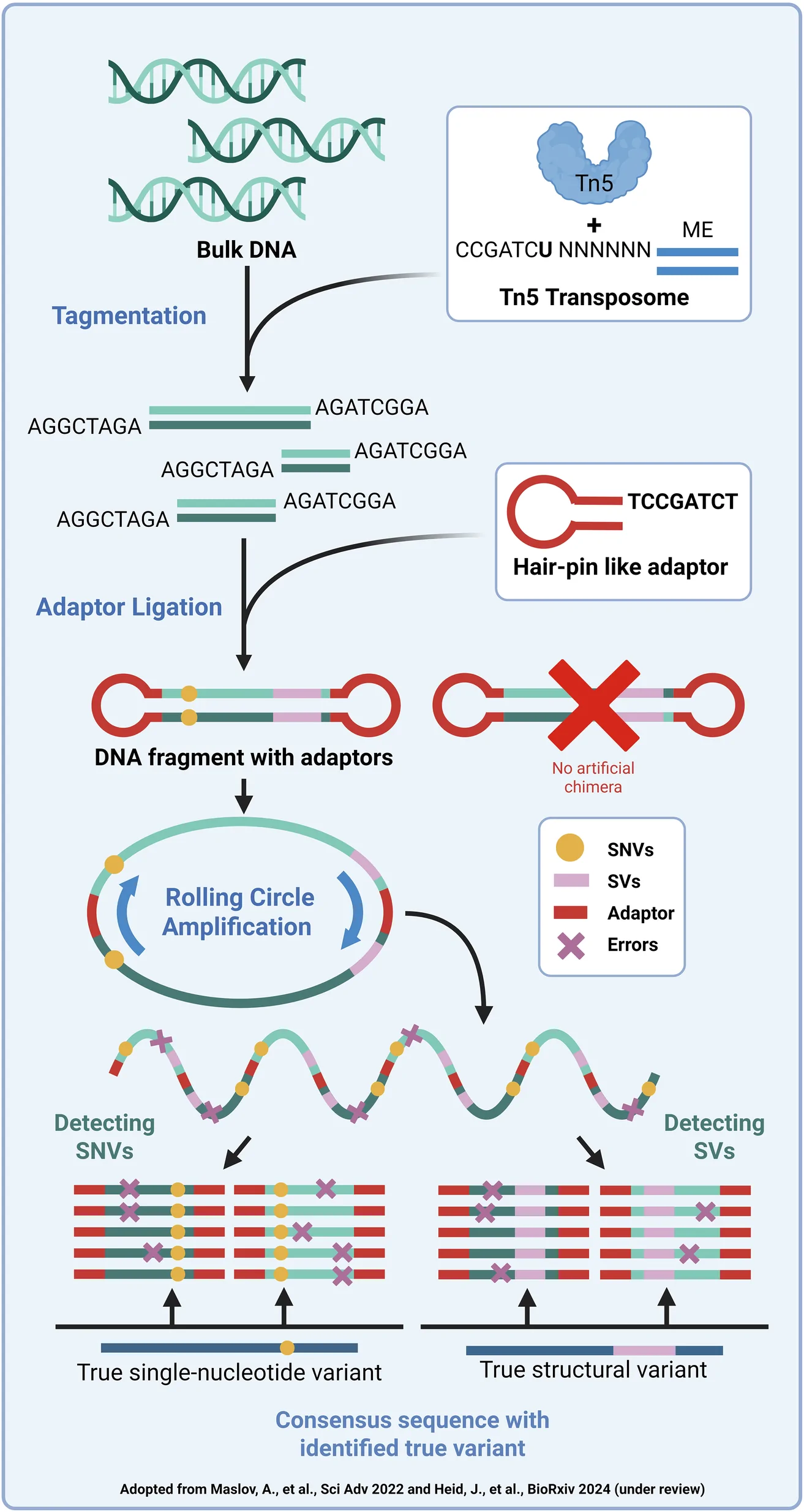
Schematic depiction of single-molecule mutation sequencing. Single-molecule mutation sequencing enables accurate detection of somatic mutations in bulk DNA samples without the high cost associated with single-cell sequencing. This technique uses rolling cycle amplification, utilizing the same DNA fragment as a template for multiple rounds of amplification, thereby facilitating the differentiation between true mutation and technical artifacts. Recently, the method has been refined to detect both single-nucleotide variants and structural variants by mitigating the formation of artificial chimeras during the ligation step. This improvement was achieved using transposomes during the fragmentation process, coupled with the incorporation of overhang sequence in the adaptor. The enhanced single-molecule mutation-seq approach offers a cost-effective and accurate alternative to study somatic mutation, potentially in large-scale studies. Figure created using BioRender.

Single-molecule assays are the novel alternative approach for detecting somatic mutations. This method, pioneered by the Loeb laboratory^38^, was initially termed “duplex sequencing.” Variants of this method have become increasingly popular owing to its ease of handling and relatively low cost. Unlike conventional NGS techniques that rely solely on one DNA strand, duplex sequencing incorporates data from both strands of the DNA molecule and thus significantly improves its accuracy for mutation calling. During the library construction, DNA fragments are “tagged” with short distinct sequences, known as unique molecular identifiers, to identify the complementary strands of each sequenced fragment. During the analysis step, these unique molecular identifiers are incorporated computationally for strand and cluster family organization. True mutations would present on both strands of the DNA molecule, whereas sequencing artifacts would typically manifest on just one strand, thus be filtered out in the analysis^38,39^. Recently, numerous enhanced iterations of duplex sequencing have been established, including BotSeqS^40^, NanoSeq^41^, and SMM-seq^42^ (Fig. 2). Notably, NanoSeq has error rates less than five per billion base pair in a single DNA molecule, which is 100 times lower than the typical somatic mutation frequencies.

It is crucial to emphasize that small insertions and deletions (INDELS) and genome structural variants (SVs) are significantly more detrimental to cellular function than SNVs. This is especially true for SVs, which can affect large parts of the genome involving multiple genes and gene regulatory sequences. Consequently, these mutations occur far less frequently and pose greater challenges for accurate quantification. Most cells that harbor such mutations are more likely to undergo apoptosis, thereby reducing their prevalence in the population studied. Many methods described so far can detect SVs to varying degrees, but with limited accuracy^43–45^. Ongoing advances of detecting SVs in single-molecule assays^42,46^ (Fig. 2) offer promising avenues for mutation analysis in somatic tissues^46^. Since the introduction of NGS, these innovations have significantly advanced our understanding of post-zygotic somatic mutations in aging and associated diseases. We now know that somatic mutations occurring during development, adulthood, and aging can lead to not only cancer but also a wide range of human diseases^47,48^.

## Somatic mutations increase with age in both humans and mice

The somatic mutation theory of aging postulates that organisms experience an increasing somatic mutation burden in their organs and tissues as they age, ultimately manifesting as age-related phenotypes. Some of the earliest evidence of somatic genome mosaicism came from cytogenetic observations of age-related Y chromosome loss (LOY) in human bone marrow cells^49^. LOY is also prevalent in peripheral blood lymphocytes, affecting more than 20% of cells in approximately half of all men by the age of 70 (ref. ^50^). Subsequent investigations using the more precise FISH-based assays expanded these initial findings, providing evidence for an age-related increase in chromosomal alterations in both mice and humans^51,52^. Using the aforementioned transgenic reporter mice, also much smaller mutation types, such as base substitutions and deletions of various sizes, became amenable for analysis as a function of age. The results of these studies confirmed that most if not all types of mutation accumulate with age but do so in a tissue-specific manner^53–55^. As expected, also pathological conditions and environmental factors significantly affect mutation susceptibility in these mice^56,57^.

Once single-cell and single-molecule approaches were widely applied, somatic mutations were found to accumulate with age in various human tissues, including brain^58^, lymphocytes^59^, liver^60^, and lung^61^. In B lymphocytes from healthy humans, the mutation burden is less than 500 SNVs per cell at birth. This number climbs considerably with age at a rate of approximately 25 SNVs/cell/year, exceeding 3,000 SNVs per cell in centenarians^59^. Normal human neurons from the prefrontal cortex and hippocampus have less than 100 SNVs in a roughly 5-month-old donor, with the number increasing to more than 2000 SNVs in donors over 80 (~12–21 SNVs/cell/year). Comparative analysis of neurons between normal and neurodegenerative diseases revealed that neurodegeneration is closely associated with increased mutation burden exhibiting distinctive mutation signatures^58,62,63^. Normal human hepatocytes have a median SNV burden of about 1,200 per cell in 35-year-old donors, which then rises to about 4,000 per cell in subjects over 46 years, a rate of approximately 52 SNVs/cell/year. This high somatic mutation rate in liver may be related to the role of organ in detoxification, subjecting the hepatocytes to constant genotoxicity^60^. Liver hepatocytes become tetraploid and octoploid during aging, which may mitigate the impact of DNA damage^64^. In human lungs, proximal bronchial basal cells of non-smokers have 464 SNVs per cell in 11-year-old donors, which then increases to almost 3,000 per cell at age 80, a rate of roughly 28 SNVs/cell/year. This mutation rate markedly rises in smokers to about 91 SNVs/cell/ year, a threefold increase^61^.

In addition to true single-cell analysis, also its surrogate, that is, growing single cells into clones, has now been widely applied for whole-genome sequencing. As mentioned earlier, similar to a tumor mass, the single-cell-derived clones also contain all the mutations present in the original single cells. Using this technique, Yoshida and co-workers reported that in bronchial epithelial cells of non-smoking donors, somatic mutations accumulate with age at a rate of approximately 22 SNVs/cell/year. In both smokers and ex-smokers, mutation burden and mutation rate were significantly higher, mirroring the findings observed by Huang et al.^61,65^, using direct analysis of single lung cells. A study by Franco et al.^66^ showed that mutations accumulate with age in skeletal muscle satellite cells at roughly 13 SNVs/genome/year. Pre-adipocytes isolated from different locations in the kidney show varying mutation rates: cells from subcutaneous fat accumulated 18 SNVs/cell/year, whereas cells from visceral fat accumulated 27 SNVs/cell/year^67^. This demonstrates location-dependent variation in mutation rate within cell types. In hematopoietic cells, Osorio et al.^68^ found that hematopoietic stem and progenitor cells amass about 14 SNVs/cell/year, resulting in about 450 SNVs per cell in 26-year-old donors and 1,000 SNVs in the 60-year-olds, slower than most differentiated cells. Interestingly, Machado et al.^69^ observed that clones from memory T and B lymphocytes have elevated mutation rates, 25 and 17 SNVs/cell/year, respectively, compared with their naive counterparts, 22 and 15 SNVs/cell/year. This has also been found in another study on B lymphocytes and is a consequence of somatic hypermutation^59^.

Clonally amplified somatic mutations arise naturally in different organs and tissues with age and can be detected by high-depth sequencing. Using LCM-seq (laser-capture microdissection and low-input DNA sequencing), small clones consisting of hundreds of cells can be captured from solid tissue and subsequently sequenced^70^. Using this technique, Moore et al.^71^ analyzed mutational landscapes in 29 cell types from the same individual and determined the tissue-specific mutation rate and mutation burden. There were 14 donors in this study and their age ranged from 22 to 83 years. Among all the analyzed tissues, colonic crypts revealed the highest mutation rate at 49–56 SNVs/cell/year, whereas spermatogonia had the lowest mutation rate at 2.38 SNVs/cell/year. It is worth mentioning that in other LCM-seq studies, high mutational burden and frequency in colonic crypt were also observed^72,73^.

NanoSeq, one of the single-molecule methods introduced earlier, has been applied to study postmitotic cells across several tissues, uncovering mutational burdens and signatures. The results were essentially similar to the observations reported for the LCM studies mentioned earlier, with sperm having the lowest mutation accumulation rate of 2.5 SNVs/cell/year, whereas colonic crypts had the highest rate of 51 SNVs/cell/year. Other tissues examined in this study include neurons with a rate of ~17 SNVs/cell/year, urothelium with ~41 SNVs/cell/year, smooth muscle cells in colon and bladder with ~21 SNVs/cell/year, and granulocytes in blood with ~20 SNVs/cell/year^41^.

In summary, we now have full consensus that somatic mutations accumulate during aging in most if not all human tissues in substantial numbers, irrespective of the technique applied for mutation analysis, in full agreement with the original predictions by Failla^5^ and Szilard^6^. There are numerous elements that may affect mutation frequency in human tissues, including the nature of the tissue and its microenvironment, exogenous genotoxic stimuli, such as exposure to tobacco smoke or UV radiation, and genetic defects in DNA repair. These results fully substantiate the observations made in the transgenic animal models now more than two decades ago. It is proposed that the extreme variation of mutation rates among different tissues is likely driven by the frequency of cell division and the duration at which the cells are required to function^55^. This could explain the lower mutation rate observed in germ cells and the higher mutation tolerance observed in colonic crypts. As intestinal epithelial cells are some of the most rapidly dividing cells in the body, replication errors are more prone to accumulate in these cell types. But as the entire colonic epithelial layer is renewed after several days, colonic cells are highly disposable and can therefore tolerate a high mutation burden. Indeed, as we show subsequently, somatic mutation burden inversely correlates with the functional lifespan of a cell.

## Somatic mutation rate inversely correlates with the functional lifespan of cells

**Fig. 3:**
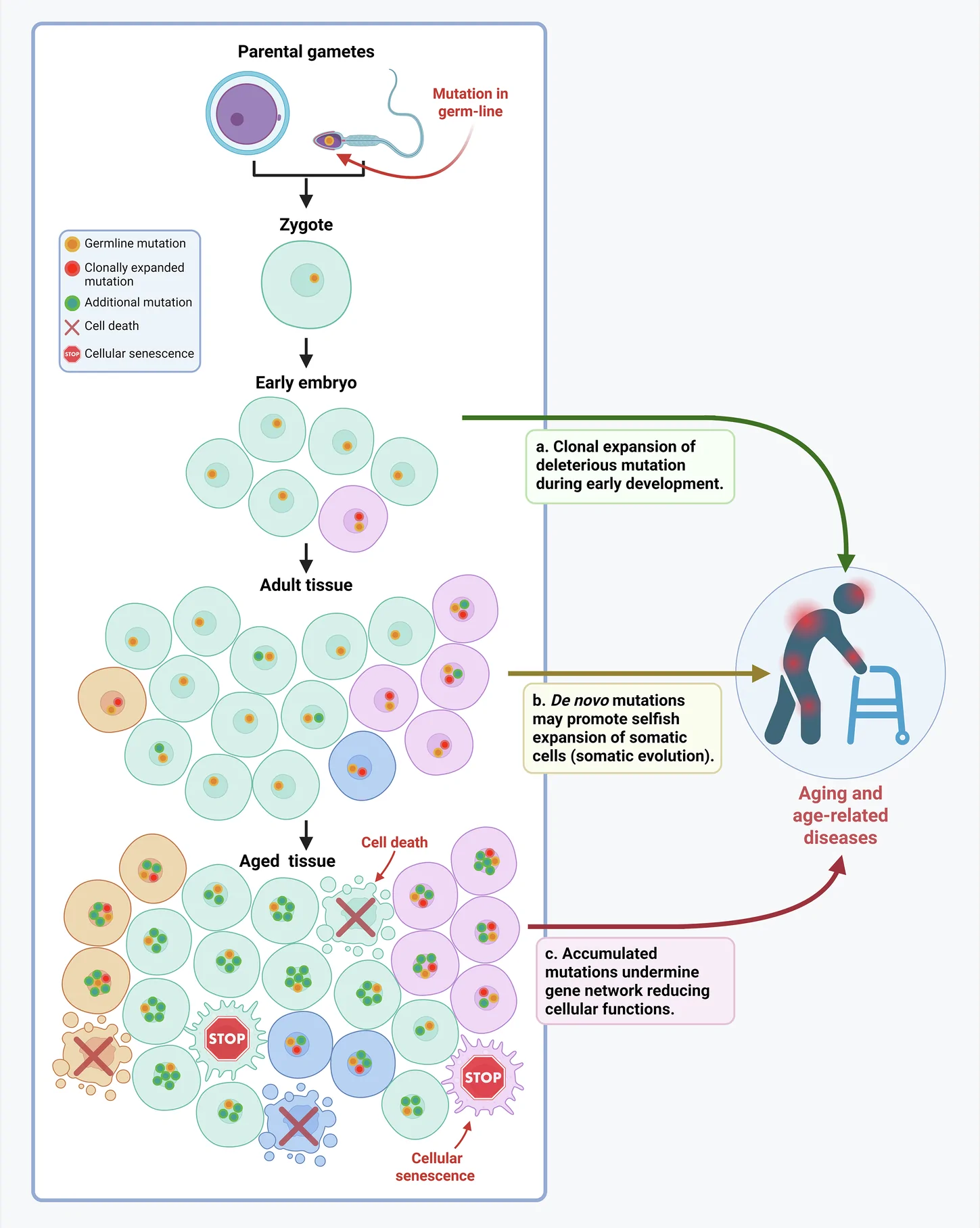
Accumulation of de novo mutations gives rise to somatic genome mosaicism. De Novo mutations emerge across the entire developmental continuum and lifespan of an organism. Germline mutations in the parental gametes are inherited and present in all the cells of the offspring (orange dots). Mutations acquired over the lifetime (green dots) are stochastic and unique to each individual cell. **a**, Nonetheless, mutations, occurring during development, adulthood, and aging (red dots) can expand clonally, eventually comprising a substantial proportion of the tissue (pink cells). Should these mutations prove deleterious, their adverse effect may parallel those of harmful germline mutation, which frequently precipitates genetic disorders. **b**, Somatic cells in adult tissues may participate in competitive interactions (orange and blue cells) during the process of somatic evolution. Cells that acquire selfish or advantageous mutations often undergo significant clonal expansion, forming major clonal lineages that perturb the homeostasis of the tissue niche, promoting carcinogenesis and various age-related pathologies. **c**, During aging, somatic mutations accumulate across the genome, including regions that may participate in critical signaling networks. The gradual erosion of network integrity potentially culminates in the attenuation of signals essential for cellular homeostasis. The diminished signaling network can subsequently result in a cascade of cellular dysfunction, contributing to the aging phenotype. SNV single-nucleotide variant, SV structural variant. Figure created using BioRender.

If somatic mutations are important as a causal factor in aging, one would expect that the mutation rate has been adapted to the time required for a particular cell type to maintain the sequence integrity of its genome. In this respect, two predictions can be made. First, germ cells are likely to have a much better quality of genome maintenance than somatic cells. Indeed, the primary distinction between somatic and germline mutations is their potential for heritability. Mutations in the soma do not transmit to the offspring (Fig. 3, green and red dots). Somatic cells are only needed for one lifetime, a concept that has been termed “disposable soma”^74^. However, germline mutations can produce gametes that pass on mutations to future generations (Fig. 3, orange dots). Germline mutations serve as the primary source of genetic diversity, providing the variation upon which natural selection operates, driving evolutionary processes. Although the mutation rate inherently must remain above zero to maintain evolutionary potential, deleterious mutation can also exert fatal outcomes on species preservation and reproductive fitness. Indeed, as mentioned earlier, germline mutations are responsible for a myriad of human heritable diseases, many of which already show in early life as developmental disorders. Many more may confer or contribute to aging-related diseases as there is little or no selection against mutations with late-life effects.

Before the emergence of methods to assess somatic mutation, it was generally assumed that somatic mutation and germline mutation have similar mutation rates. However, with the emergence of the advanced sequencing methods described earlier, it became possible to directly compare germline and somatic mutation rates. In both mice and humans, the latter was found to be 10–100-fold higher than the former^75^. This is in keeping with the generally low mutation burden found in germ tissues. As expected, the rate of mutation was the lowest in spermatogonia from which sperm is generated and most genetic variation in the human population originates^71^. There is also evidence that somatic mutation rate in stem cells is lower than that in fully differentiated cells^60,76,77^; this is yet another example of an inverse correlation of mutation rate with the necessary time during which a cell needs to maintain a pristine genome.

A second functional phenotype inversely correlated with somatic mutation rate is species-specific maximum lifespan. This is a corollary of the early idea that DNA repair capacity is directly correlated with species lifespan, something for which there is indeed evidence^78^. Early studies on neutral mutation rates of DNA sequences during evolution, estimated from interspecies DNA sequence differences, indicated much faster rates in rodent lineages when compared with primates. This was ascribed to superior DNA repair in the longer-lived primates^79^. In primates, the rate of evolutionary change was found to slow down during evolution of the longest-lived primates, such as chimpanzees and humans, that is, the hominoid slowdown^80^. A possible explanation proffered was that DNA repair capacity increased at pace with the evolution of longer-lived species. With the emergence of advanced single-cell sequencing methods, it became possible to directly compare somatic mutation rates between cells from short-lived and long-lived species. In keeping with the disposable soma theory of aging, which states that organisms balance their energy allocation between reproduction and somatic maintenance^74^, short-lived species would be predicted to spend less energy on genome maintenance than long-lived species. This prediction was proved correct when cells from short-lived rodents, such as mice, were demonstrated to have a higher mutagen-induced mutation burden than cells from long-lived rodent species such as naked mole rats^81^. Shortly thereafter, an inverse correlation between age-related somatic mutation rate in colonic crypts and species-specific lifespan was indeed found^73^.

Hence, the above seems to indicate that the amount of energy cells allocated to genome maintenance depends on the time they need to function optimally. This explains why mutation rates are lower in germ cells, which are immortal, than in differentiated cells, which only need to function for a lifetime or less. Also stem cells, which need to replace differentiated cells, appear to have reduced somatic mutation rates when compared with differentiated cells. This does not mean that somatic mutation rates can evolve to zero. Indeed, maintaining a pristine genome indefinitely has its own fitness cost and is unnecessary because of ongoing selection against heavily mutated cells. For example, only a fraction of germ cells leads to successful conception and healthy offspring, most likely due to selection against heavily mutated cells^82,83^.

## Progeroid syndromes and somatic mutation rates

Human genetic syndromes showing multiple symptoms of aging prematurely are known since the nineteenth century^84^. It was only much later that such syndromes were found to be almost exclusively due to heritable defects in genome maintenance (Table 1). The best example is Werner syndrome (WS), the canonical adult-onset syndrome of premature aging. WS is an autosomal recessive disorder resulting from mutations in RECQ3, a member of the RecQ family of DNA helicases. Premature aging symptoms develop from the third decade of life and include skin atrophy, loss and graying of hair, type 2 diabetes, osteoporosis, cataracts, gonadal atrophy, severe forms of arteriosclerosis, and cancer. Patients with WS die in their 1950s, mostly from cancer^85^. The RecQ helicase family has crucial roles in DNA replication, telomere maintenance, and DNA repair mechanisms^86^. Using the HPRT reporter assay, Fukuchi et al.^87^ have indeed observed increased mutational frequency, characterizing many of the mutations as large deletions. Later, Werner cells were found to exhibit more frequent telomere shortening and loss, visualized by FISH probes^88,89^. Accordingly, chromosomal aberrations such as translocations also appeared to be more prevalent in Werner cells, using both Giemsa staining and Spectral Karyotyping^90,91^. Collectively, this indicates increased genome instability in WS. It should be noted that not all normal aging symptoms are accelerated in WS. For example, patients with WS do not show symptoms of neurodegeneration, a major phenotype of normal aging; hence, the term “segmental progeroid syndromes”^92^.

**Table 1:** Progeroid disease with corresponding age-associated symptoms and affected genome maintenance pathways.

Disease	Affected genome maintenance pathway	Mutated genes	Life expectancy	Age-associated symptoms	Refs.
**Hutchinson–Gilford progeria syndrome**	Chromatin organization	*LMNA*	~14.6 years (range: 6–27 years)	Alopecia, atherosclerosis, arthritis, cardiovascular disease, lipodystrophy, osteoporosis, skin aging, and atrophy	Kudlow et al. (2007); Liu et al. (2005)
**Cerebroretinal microangiopathy with calcification and cysts (Coats plus syndrome)**	Telomere maintenance, protection from DNA replication stress	*CTC1*	Death occurs between 2 and 30 years old	Prenatal and postnatal growth defects, retinal vascular abnormalities, osteoporosis, thin skin, graying of hair, anemia, bone marrow failure, microangiopathy, calcification, subcortical cyst, higher risk of cancer, and leukoencephalopathy	Anderson et al. (2012); Chen et al. (2013)
**Nestor–Guillermo progeria syndrome**	Chromatin organization	*BANF1*	Not well-established	Alopecia, atherosclerosis, arthritis, cardiovascular disease, lipodystrophy, osteoporosis, and pulmonary hypertension	Cabanillas et al. (2011); Loi et al. (2016)
**Bloom syndrome**	DNA replication and recombination	*BLM*	~27 years (median)	Diabetes, pulmonary disease, increased risk of cancer, rash on face due to sun exposure, immune deficiency, and dyslipidemia	Hanada and Hickson (2007); De Renty and Ellis (2017)
**Nijmegen breakage syndrome (NBS)**	Double-strand break (DSB) repair, telomere stability, oxidative stress	*NBS1*	~30–40 years, lack of conclusive report	Microcephaly, growth retardation, mental retardation, primary ovarian failure, immunodeficiency, and increased incidence of cancer	Varon et al. (1998); Zhu et al. (2001)
**Werner syndrome**	Telomeric maintenance and replication stress	*WRN*	~54 years	Alopecia, atherosclerosis, arthritis, cardiovascular disease, cataracts, diabetes, sarcopenia, and increased risks of cancer	Kudlow et al. (2007); Sugimoto (2014); Oshima et al. (1996)
**Rothmund–Thomson syndrome**	DNA replication initiation	*RECQL4*	Near-normal if cancer-free	Alopecia, cataracts, osteoporosis, skin atrophy, and increased risks of cancer	Croteau et al. (2012); Ghosh et al. (2012)
**Ataxia telangiectasia**	DNA damage response	*ATM*	~19–25 years (median)	Premature bone marrow exhaustion, diabetes, and neurodegeneration	Rothblum-Oviatt et al. (2016); Shiloh et al. (2017)
**XFE progeroid syndrome**	Nucleotide excision repair (NER), ICL, and DSB repair	*ERCC4*	~16 years (case reports)	Anemia, cardiovascular disease, kidney disease, neurodegeneration, osteoporosis, sarcopenia, sensory loss, and skin atrophy	Niedernhofer et al. (2006)
**Xeroderma pigmentosum**	NER and translesion DNA synthesis	*XPA-G, XPV*	~37 years, if without neurodegeneration	Premature skin photoaging, neurodegeneration, and increased incidence of skin cancer	Lehmann et al. (2011); Kraemer and DiGiovanna (2015)
**Trichothiodystrophy**	Transcription-coupled NER	*TTDA*, *TTDN1*, *XPB*, *XPD*	~3 years, in severe case	Premature bone marrow exhaustion and increased risk of cancer	Faghri et al. (2008); De Boer et al. (2002)
**Cockayne syndrome**	Transcription-coupled NER	*CSA*, *CSB*, *XPD*, *XPG*	~10–20 years	Ataxia, cataracts, muscle atrophy, and neurodegeneration	Nance and Berry, (1992); Wilson et al. (2016)
**Fanconi anemia**	ICL repair	*FANCA-FANCW*	20–30 years	Premature bone marrow exhaustion and increased risk of cancer	Ceccaldi et al. (2016) Nalepa and Clapp (2018)
**Mandibular hypoplasia, deafness, progeroid features, lipodystrophy syndrome**	Post-replication repair and translesion DNA synthesis	*POLD1*	Not well-established, patient reaches adulthood	Diabetes, lipodystrophy, osteoporosis, steatosis, sensory loss	Weedon et al. (2013)
**Alpers–Huttenlocher syndrome**	Mitochondrial DNA replication and repair	*POLG1*	<4 years, post-symptom onset	Progressive neurodegeneration and liver disease	Nguyen et al. (2006)
**Ruijs–Aalfs syndrome**	Protein–DNA crosslink repair	*SPRTN*	Death occurs in adolescence to early adulthood	Alopecia, atherosclerosis, cataracts, diabetes, premature graying of hair, osteoporosis, sarcopenia, and increased risk of cancer	Weickert et al. (2023); Lessel et al. (2014)

There are other human progeroid syndromes based on genetic defects in RecQ genes or in other DNA repair genes as well. For example, xeroderma pigmentosum^93^ and ataxia telangiectasia^94^, based on a genetic defect in nucleotide excision repair and the DNA damage response gene ATM, respectively, have now been recognized as genuine progeroid syndromes^93,94^. In an XPA knockout mouse model, a significantly higher somatic mutation burden was observed in liver and kidney than in control mice^95^. In the brain of patients with ataxia telangiectasia, increased levels of aneuploidy were found when compared with the control^96^. Also DS is now recognized as a premature aging syndrome owing to the prevalent incidence of premature neurodegeneration and Alzheimer disease, affecting ~75% of the DS population at the age of 60 (ref. ^97^). Somatic mutation rate in fetal hematopoietic stem and progenitor cells of patients with DS has been found to be increased, which was suggested to contribute to the increased risk of leukemia in this progeroid syndrome^98^. There is no information on genome instability in the brain cells of patients with DS.

The second best known segmental progeroid syndrome is Hutchinson–Gilford Progeria Syndrome (HGPS)^84,99^. HGPS is a developmental disease and has initially not been associated with DNA repair defects. However, the genetic defect in this disease affects lamin, a crucial component of the nuclear lamina, resulting in malformed nuclei^100^. In human skin fibroblasts, HGPS cells have altered histone marks (H3K27me3) in heterochromatin, disrupting normal chromatin organization^101^. Cytogenic comparisons of immortalized cells derived from HGPS and normal cells have also revealed a significant increase in chromosomal abnormalities^102,103^. Liu et al.^104^ demonstrated that the accumulation of dysfunctional lamin A in HGPS can impede the recruitment of crucial DNA repair proteins, including 53BP1 and Rad51, thereby compromising DNA repair and heightening sensitivity to genotoxic agents.

The idea that premature aging is associated with genetic defects in DNA repair and increased genome instability gained strong support when DNA repair defects engineered in the mouse were found to be associated with multiple symptoms of premature aging^105–108^. Since to our knowledge there are no other gene families in which defects lead to premature aging, the exclusive connection of premature aging with DNA repair strongly suggests a role of genome instability, a primary hallmark of aging, as a basic mechanism of age-related cellular degeneration and death.

## Functional consequences of somatic mutations

From the above the question that now arises is whether somatic mutations can ever accumulate to levels causing age-related functional loss and increased incidence of disease. Indeed, the clearest example of an age-related illness caused by somatic mutations is cancer. Cancer is known to be caused by somatic mutations, and it is tempting to suggest that this is part of a more general causal role of mutations in aging. However, other age-related changes, most notably immunosenescence, could have a major role here too.

Other age-related pathology has also been shown to be caused by clonally amplified somatic mutations^109,110^. Some best-known examples are somatic counterparts of Mendelian genetic diseases, including neurofibromatosis 1, Proteus syndrome, and McCune–Albright syndrome^48,111^. Numerous if not all cases in these diseases are caused by somatic mutations that occurred in early embryonic development, resulting in a significant portion of tissue harboring the mutation. The disease then becomes manifest with the progression of aging. At times, genetic disease can also be caused by the combined effect of germline and somatic mutations. Such is the case in autosomal dominant polycystic kidney disease (ADPKD). The individual first inherits an inactivated allele of ADPKD2 gene through the germline, after which the disease only manifests after acquiring the second, somatic mutation. The cysts formed in this case are caused by the clonal expansion from the second somatic mutation^112^.

A major outstanding question is whether increased numbers of somatic mutations can collectively cause functional decline of organs or tissues. In this respect, it is worth reiterating that the overwhelming majority of somatic mutations are either neutral or demonstrably deleterious^20^. Increased somatic mutations have been implicated in the increased cell-to-cell heterogeneity in gene transcript levels during aging^113^. Age-related transcriptional noise (as this is called) has now been demonstrated in multiple human and mouse tissues^114^. It has been hypothesized that somatic mutations in gene regulatory sequences could be causally related to this increase in transcriptional noise and contribute to a general loss of gene regulation with age^22,115^. If this would occur in a substantial fraction of cells in a tissue, it could causally contribute to the observed age-related decline in function.

Under highly specific circumstances, somatic mutations can sometimes confer functional advantages. It is essential to define what “beneficial” means in this context. The adaptive worth of any given mutation is not an intrinsic property but is instead contingent on temporal, cellular, and organismal factors; when does the mutation arise, whether the benefit accrues to the cell, the tissue, or the organism, and whether there exists active selection pressure for the resulting phenotype^116^. The clearest example of beneficial somatic mutation occurs within the adaptive immune system, in which the process is tightly regulated. During B cell maturation, somatic hypermutation is selectively activated within the variable regions of immunoglobulin genes at a rate of roughly one mutation per 1,000 base pairs, orders of magnitude higher than baseline somatic mutation rate^117,118^. This targeted mutagenesis generates a highly diverse repertoire of antibody variants, from which cells expressing high-affinity antigen-binding sequences are then positively selected through affinity maturation in germinal centers^118^. Here, the benefit for the organism is unambiguous. Of note, the beneficial somatic mutations in this case do not arise spontaneously but require a precisely coordinated mutational mechanism coupled with stringent, active selection to produce a functional outcome.

Using whole-exome and ultra-deep targeted sequencing, Zhu et al.^119^ identified recurrent mutations in four genes (*PLD1*, *KMT2D*, *PPARGC1B*, and *ARID1A*) in non-malignant human liver tissue. To functionally validate these targets, CRISPR screen knockout of these genes in mice confirmed that loss-of-function mutations imparted a survival advantage to hepatocytes during liver regeneration. Critically, these mutations were not associated with any liver cancer^119^, suggesting that their selective advantage operates within the bounds of normal tissue physiology. It is possible that other clonally amplified somatic mutations will be found. For now, beneficial effects of random somatic mutations for the organism seem rare. However, selfish mutations that provide a growth advantage to a cell but are not carcinogenic have been demonstrated in the form of clonal hematopoiesis (CH).

In CH, beneficial somatic mutation and fitness become considerably more complex and dynamic. Mutations in the *DNMT3A*, *TET2*, and *ASXL1* (DTA) genes represent the most frequently observed alterations in CH^120^, and many such variants skew hematopoietic stem cell fate away from differentiation and toward self-renewal, granting a cell-intrinsic proliferative advantage. Although this is “beneficial” from the perspective of the mutant clone, CH is robustly associated with myriads of pathologies, including hematological malignancy and cardiovascular diseases. Intriguingly, CH of indeterminate potential^121^, a subset of CH defined by the presence of DTA mutations in the absence of overt hematological disease, has been associated with unexpected neuroprotective effects. Bouzid et al.^122^ reported that CH of indeterminate potential carriers exhibited a significantly reduced risk of Alzheimer disease. It was shown that mutations detected in peripheral blood were also identified in microglial cells, raising the possibility that clonally expanded mutant immune cells within the central nervous system may attenuate neuroinflammatory pathways underlying AD. All together, these findings highlight the nuance and dynamics that must be considered on the topic of beneficial somatic mutation.

## Conclusions and future directions

Since its conception, the somatic mutation theory of aging has catalyzed significant advances in our understanding of aging. It has driven the development of new technology and new animal models to begin testing the possible causal role of somatic mutations in the aging process. Mouse models harboring reporter genes that can be rescued from their integrated state and studied in *Escherichia coli* for mutations arising in the animal during aging first demonstrated that mutations accumulate during aging in a tissue-specific manner^53^. More recently, single-cell and single-molecule sequencing-based assays allowed to confirm the mouse results in humans^55^. Meanwhile, deep sequencing demonstrated that age-related genome mosaicism is a general phenomenon in humans and often a cause of disease^109^. The further characterization of somatic mutations across tissues has yielded age-specific, tissue-specific, and pathology-specific mutational landscapes, suggesting potential mechanistic insights into disease progression and natural cellular function decline. Nevertheless, several technical and conceptual issues remain unresolved.

Although it is now considerably more feasible to quantify SNVs and small INDELs as somatic mutations in tissues, it is still notably difficult to accurately measure the much less frequent genome SVs. SVs are generally much more impactful than SNVs or INDELS, potentially disrupting multiple genes, gene networks, and regulatory elements. The large size, intricate structural complexity, and frequent localization within repetitive genomic region render SVs particularly challenging to detect^123^. Single-cell techniques and bulk DNA analysis have been explored to detect SVs with notable drawbacks, including the introduction of artifacts in the form of chimeric sequences during library preparation^43^. Especially, the use of long-read sequencing may help to resolve this issue^124^.

A second challenge remains the establishment of a cause-and-effect relationship between random somatic mutations and age-related disease risk and functional decline. The by now well-documented observations that somatic mutations accumulate with age in most if not all tissues in fairly large numbers and the observed inverse correlation of somatic mutation rate with species-specific lifespan both point to a causal role for a loss of genome sequence integrity in aging. However, the underlying mechanisms linking somatic mutation to aging phenotypes remain poorly understood. The best evidence for a causal relationship between accumulating mutations and aging is still the exponential increase of cancer risk with age^125^.

Finally, if somatic mutations would be demonstrated as a major cause of aging, can we develop interventions to slow down mutation accumulation or reduce its adverse effects? The most obvious way to do this is by improving DNA repair. This has been notoriously difficult owing to the complexity of the network of DNA repair systems. Indeed, simply upregulating individual DNA repair factors would likely lead to loss of stoichiometry with potentially toxic effects. One recently discovered possibility to upregulate all or most DNA repair pathways collectively is by inhibiting the *dimerization partner, RB-like, E2F and multi-vulval class B* (DREAM) complex, which is known to have a unifying role in the cell cycle^126,127^. It has been shown that the DREAM repressor complex curbs DNA-repair capacities in somatic tissues of *Caenorhabditis elegans*^128^. Interventions that inhibit DREAM and therefore upregulate DNA repair could have a future role in the now rapidly expanding arsenal of gerotherapeutics^129^.

## References

[1] Dev, H. The origin of species by mutation. *Science* **15**, 721–729 (1902). 17793609 10.1126/science.15.384.721

[2] Boveri, T. Zur frage der entstehung maligner tumoren. Fischer; 1914.

[3] Morgan TH, Bridges CB & Sturtevant AH. The origin of gynandromorphs. Carnegie Inst.; 1919.

[4] Johannsen, W. The genotype conception of heredity. *Am Nat* **45**, 129–159 (1911). 10.1093/ije/dyu063 PMC4258772 24691957

[5] Failla, G. The aging process and cancerogenesis. *Ann N Y Acad Sci.* **71**, 1124–1140 (1958). 13583876 10.1111/j.1749-6632.1958.tb46828.x

[6] Szilard, L. On the nature of the aging process. *Proc Natl Acad Sci USA* **45**, 30–45 (1959). 16590351 10.1073/pnas.45.1.30 PMC222509

[7] Jacobs, P. A., Brunton, M. & BROWN, W. C. Cytogenetic studies in leucocytes on the general population: subjects of ages 65 years and more. *Ann Hum Genet* **27**, 353–362 (1963). 10.1111/j.1469-1809.1963.tb01532.x 14175200

[8] Nowell, P. C. The clonal evolution of tumor cell populations: acquired genetic lability permits stepwise selection of variant sublines and underlies tumor progression. *Science* **194**, 23–28 (1976). 959840 10.1126/science.959840

[9] Lindahl, T. Instability and decay of the primary structure of DNA. *Nature* **362**, 709–715 (1993). 8469282 10.1038/362709a0

[10] De Bont, R. & van Larebeke, N. Endogenous DNA damage in humans: a review of quantitative data. *Mutagenesis* **19**, 169–185 (2004). 15123782 10.1093/mutage/geh025

[11] Huang, J.-C., Hsu, D. S., Kazantsev, A. & Sancar, A. Substrate spectrum of human excinuclease: repair of abasic sites, methylated bases, mismatches, and bulky adducts. *Proc Natl Acad Sci USA* **91**, 12213–12217 (1994). 7991608 10.1073/pnas.91.25.12213 PMC45407

[12] Brenneisen, P., Sies, H. & Scharffetter-Kochanek, K. Ultraviolet-B irradiation and matrix metalloproteinases: from induction via signaling to initial events. *Ann N Y Acad Sci.* **973**, 31–43 (2002). 12485830 10.1111/j.1749-6632.2002.tb04602.x

[13] Birnboim, H. & Jevcak, J. Fluorometric method for rapid detection of DNA strand breaks in human white blood cells produced by low doses of radiation. *Cancer Res* **41**, 1889–1892 (1981). 7214357

[14] Hoeijmakers, J. H. DNA damage, aging, and cancer. *N Engl J Med.* **361**, 1475–1485 (2009). 19812404 10.1056/NEJMra0804615

[15] Liu, Y. et al. Coordination of steps in single-nucleotide base excision repair mediated by apurinic/apyrimidinic endonuclease 1 and DNA polymerase β. *J Biol Chem.* **282**, 13532–13541 (2007). 17355977 10.1074/jbc.M611295200 PMC2366199

[16] Schärer, O. D. Nucleotide excision repair in eukaryotes. *Cold Spring Harbor Perspect Biol* **5**, a012609 (2013). 10.1101/cshperspect.a012609 PMC3783044 24086042

[17] Iyer, R. R., Pluciennik, A., Burdett, V. & Modrich, P. L. DNA mismatch repair: functions and mechanisms. *Chem Rev.* **106**, 302–323 (2006). 16464007 10.1021/cr0404794

[18] Haber, J. E., Ira, G., Malkova, A. & Sugawara, N. Repairing a double-strand chromosome break by homologous recombination: revisiting Robin Holliday’s model. *Philos Trans R Soc Lond. Ser B Biol Sci.* **359**, 79–86 (2004). 15065659 10.1098/rstb.2003.1367 PMC1693306

[19] Beck, B. D., Lee, S.-S., Williamson, E., Hromas, R. A. & Lee, S.-H. Biochemical characterization of Metnase’s endonuclease activity and its role in NHEJ repair. *Biochemistry.* **50**, 4360–4370 (2011). 21491884 10.1021/bi200333k PMC3388547

[20] Lee CE, Singleton KS, Wallin M, Faundez V. Rare genetic diseases: nature’s experiments on human development. *iScience* **23**, 101123 (2020). 10.1016/j.isci.2020.101123 PMC7229282 32422592

[21] Wood, K. A. & Goriely, A. The impact of paternal age on new mutations and disease in the next generation. *Fertil Steril* **118**, 1001–1012 (2022). 36351856 10.1016/j.fertnstert.2022.10.017 PMC10909733

[22] Vijg, J. & Dong, X. Pathogenic mechanisms of somatic mutation and genome mosaicism in aging. *Cell* **182**, 12–23 (2020). 32649873 10.1016/j.cell.2020.06.024 PMC7354350

[23] Jacobs, P. A., Court Brown, W. M. & Doll, R. Distribution of human chromosome counts in relation to age. *Nature* **191**, 1178–1180 (1961). 13718573 10.1038/1911178a0

[24] Jacobs, P. A., Brunton, M. & Brown, W. M. Cytogenetic studies in leucocytes on the general population: subjects of ages 65 years and more. *Ann Hum Genet* **27**, 353–365 (1964). 14175200 10.1111/j.1469-1809.1963.tb01532.x

[25] Andriani, G. A. et al. A direct comparison of interphase FISH versus low-coverage single cell sequencing to detect aneuploidy reveals respective strengths and weaknesses. *Sci Rep.* **9**, 10508 (2019). 31324840 10.1038/s41598-019-46606-w PMC6642082

[26] Gossen, J. A. et al. Efficient rescue of integrated shuttle vectors from transgenic mice: a model for studying mutations in vivo. *Proc Natl Acad Sci USA* **86**, 7971–7975 (1989). 2530578 10.1073/pnas.86.20.7971 PMC298194

[27] Kohler, S. W. et al. Spectra of spontaneous and mutagen-induced mutations in the lacI gene in transgenic mice. *Proc Natl Acad Sci USA* **88**, 7958–7962 (1991). 1832771 10.1073/pnas.88.18.7958 PMC52424

[28] Boerrigter, M. E., Dolle, M. E., Martus, H. J., Gossen, J. A. & Vijg, J. Plasmid-based transgenic mouse model for studying in vivo mutations. *Nature* **377**, 657–659 (1995). 7566182 10.1038/377657a0 PMC12990988

[29] Gossen, J. A., de Leeuw, W. J. & Vijg, J. LacZ transgenic mouse models: their application in genetic toxicology. *Mutat Res.* **307**, 451–459 (1994). 7514719 10.1016/0027-5107(94)90256-9

[30] Blokzijl, F. et al. Tissue-specific mutation accumulation in human adult stem cells during life. *Nature* **538**, 260–264 (2016). 27698416 10.1038/nature19768 PMC5536223

[31] Nam, C. H. et al. Widespread somatic L1 retrotransposition in normal colorectal epithelium. *Nature* **617**, 540–547 (2023). 37165195 10.1038/s41586-023-06046-z PMC10191854

[32] Youk, J., Kwon, H. W., Kim, R. & Ju, Y. S. Dissecting single-cell genomes through the clonal organoid technique. *Exp Mol Med.* **53**, 1503–1511 (2021). 34663940 10.1038/s12276-021-00680-1 PMC8569207

[33] Gundry, M., Li, W., Maqbool, S. B. & Vijg, J. Direct, genome-wide assessment of DNA mutations in single cells. *Nucleic Acids Res.* **40**, 2032–2040 (2012). 22086961 10.1093/nar/gkr949 PMC3300019

[34] Zong, C., Lu, S., Chapman, A. R. & Xie, X. S. Genome-wide detection of single-nucleotide and copy-number variations of a single human cell. *Science* **338**, 1622–1626 (2012). 23258894 10.1126/science.1229164 PMC3600412

[35] Gonzalez-Pena, V. et al. Accurate genomic variant detection in single cells with primary template-directed amplification. *Proc Natl Acad Sci USA* **118**, e2024176118 (2021). 34099548 10.1073/pnas.2024176118 PMC8214697

[36] Dong, X. et al. Accurate identification of single-nucleotide variants in whole-genome-amplified single cells. *Nat Methods* **14**, 491–493 (2017). 28319112 10.1038/nmeth.4227 PMC5408311

[37] Bohrson, C. L. et al. Linked-read analysis identifies mutations in single-cell DNA-sequencing data. *Nat Genet* **51**, 749–754 (2019). 30886424 10.1038/s41588-019-0366-2 PMC6900933

[38] Schmitt, M. W. et al. Detection of ultra-rare mutations by next-generation sequencing. *Proc Natl Acad Sci USA* **109**, 14508–14513 (2012). 22853953 10.1073/pnas.1208715109 PMC3437896

[39] Schmitt, M. W. et al. Sequencing small genomic targets with high efficiency and extreme accuracy. *Nat Methods* **12**, 423–425 (2015). 25849638 10.1038/nmeth.3351 PMC4414912

[40] Hoang, M. L. et al. Genome-wide quantification of rare somatic mutations in normal human tissues using massively parallel sequencing. *Proc Natl Acad Sci USA* **113**, 9846–9851 (2016). 27528664 10.1073/pnas.1607794113 PMC5024639

[41] Abascal, F. et al. Somatic mutation landscapes at single-molecule resolution. *Nature* **593**, 405–410 (2021). 33911282 10.1038/s41586-021-03477-4

[42] Maslov, A. Y. et al. Single-molecule, quantitative detection of low-abundance somatic mutations by high-throughput sequencing. *Sci Adv.* **8**, eabm3259 (2022). 35394831 10.1126/sciadv.abm3259 PMC8993124

[43] Maslov, A. Y., Quispe-Tintaya, W., Gorbacheva, T., White, R. R. & Vijg, J. High-throughput sequencing in mutation detection: a new generation of genotoxicity tests? *Mutat Res.* **776**, 136–143 (2015). 25934519 10.1016/j.mrfmmm.2015.03.014 PMC4532622

[44] Laufer, V. A., Glover, T. W. & Wilson, T. E. Applications of advanced technologies for detecting genomic structural variation. *Mutat Res.* **792**, 108475 (2023). 10.1016/j.mrrev.2023.108475 PMC10792551 37931775

[45] Collins RL, Talkowski ME. Diversity and consequences of structural variation in the human genome. *Nat Rev Genet* **26**, 1–20 (2025). 10.1038/s41576-024-00808-9 39838028

[46] Heid J, et al. Detection of genome structural variation in normal cells and tissues by single molecule sequencing. Preprint at *bioRxiv* (2024).

[47] Li, C. & Williams, S. M. Human somatic variation: it’s not just for cancer anymore. *Curr Genet Med Rep.* **1**, 212–218 (2013).

[48] Erickson, R. P. Somatic gene mutation and human disease other than cancer: an update. *Mutat Res* **705**, 96–106 (2010). 20399892 10.1016/j.mrrev.2010.04.002

[49] Pierre, R. V. & Hoagland, H. C. Age-associated aneuploidy: loss of Y chromosome from human bone marrow cells with aging. *Cancer* **30**, 889–894 (1972). 4116908 10.1002/1097-0142(197210)30:4<889::aid-cncr2820300405>3.0.co;2-1

[50] Thompson, D. J. et al. Genetic predisposition to mosaic Y chromosome loss in blood. *Nature* **575**, 652–657 (2019). 31748747 10.1038/s41586-019-1765-3 PMC6887549

[51] Tucker, J. D., Spruill, M. D., Ramsey, M. J., Director, A. D. & Nath, J. Frequency of spontaneous chromosome aberrations in mice: effects of age. *Mutat Res.* **425**, 135–141 (1999). 10082924 10.1016/s0027-5107(99)00036-6

[52] Ramsey, M. J. et al. The effects of age and lifestyle factors on the accumulation of cytogenetic damage as measured by chromosome painting. *Mutat Res* **338**, 95–106 (1995). 7565886 10.1016/0921-8734(95)00015-x

[53] Dollé, M. E. et al. Rapid accumulation of genome rearrangements in liver but not in brain of old mice. *Nat Genet* **17**, 431–434 (1997). 9398844 10.1038/ng1297-431

[54] Vijg, J. & Dollé, M. E. Large genome rearrangements as a primary cause of aging. *Mech Ageing Dev* **123**, 907–915 (2002). 12044939 10.1016/s0047-6374(02)00028-3

[55] Ren, P., Dong, X. & Vijg, J. Age-related somatic mutation burden in human tissues. *Front Aging* **3**, 1018119 (2022). 36213345 10.3389/fragi.2022.1018119 PMC9534562

[56] Giese, H., Dollé, M. E., Hezel, A., Van Steeg, H. & Vijg, J. Accelerated accumulation of somatic mutations in mice deficient in the nucleotide excision repair gene XPA. *Oncogene* **18**, 1257–1260 (1999). 10022133 10.1038/sj.onc.1202404

[57] Beal, M. A. et al. Chemically induced mutations in a MutaMouse reporter gene inform mechanisms underlying human cancer mutational signatures. *Commun Biol* **3**, 438 (2020). 32796912 10.1038/s42003-020-01174-y PMC7429849

[58] Lodato, M. A. et al. Aging and neurodegeneration are associated with increased mutations in single human neurons. *Science* **359**, 555–559 (2018). 29217584 10.1126/science.aao4426 PMC5831169

[59] Zhang, L. et al. Single-cell whole-genome sequencing reveals the functional landscape of somatic mutations in B lymphocytes across the human lifespan. *Proc Natl Acad Sci USA* **116**, 9014–9019 (2019). 30992375 10.1073/pnas.1902510116 PMC6500118

[60] Brazhnik, K. et al. Single-cell analysis reveals different age-related somatic mutation profiles between stem and differentiated cells in human liver. *Sci Adv.* **6**, eaax2659 (2020). 32064334 10.1126/sciadv.aax2659 PMC6994209

[61] Huang, Z. et al. Single-cell analysis of somatic mutations in human bronchial epithelial cells in relation to aging and smoking. *Nat Genet* **54**, 492–498 (2022). 35410377 10.1038/s41588-022-01035-w PMC9844147

[62] Lodato, M. A. et al. Somatic mutation in single human neurons tracks developmental and transcriptional history. *Science* **350**, 94–98 (2015). 26430121 10.1126/science.aab1785 PMC4664477

[63] Miller, M. B. et al. Somatic genomic changes in single Alzheimer’s disease neurons. *Nature* **604**, 714–722 (2022). 35444284 10.1038/s41586-022-04640-1 PMC9357465

[64] Hayashi, K. et al. Polyploidy mitigates the impact of DNA damage while simultaneously bearing its burden. *Cell Death Discov.* **10**, 436 (2024). 39397009 10.1038/s41420-024-02206-w PMC11471775

[65] Gowers, K. et al. Tobacco exposure and somatic mutations in normal human bronchial epithelium. *Am. J. Respir. Crit. Care Med.* **201**, A4090 (2020).

[66] Franco, I. et al. Somatic mutagenesis in satellite cells associates with human skeletal muscle aging. *Nat Commun.* **9**, 800 (2018). 29476074 10.1038/s41467-018-03244-6 PMC5824957

[67] Franco, I. et al. Whole genome DNA sequencing provides an atlas of somatic mutagenesis in healthy human cells and identifies a tumor-prone cell type. *Genome Biol.* **20**, 1–22 (2019). 31849330 10.1186/s13059-019-1892-z PMC6918713

[68] Osorio, F. G. et al. Somatic mutations reveal lineage relationships and age-related mutagenesis in human hematopoiesis. *Cell Rep* **25**, 2308–2316.e4 (2018). 30485801 10.1016/j.celrep.2018.11.014 PMC6289083

[69] Machado, H. E. et al. Diverse mutational landscapes in human lymphocytes. *Nature* **608**, 724–732 (2022). 35948631 10.1038/s41586-022-05072-7 PMC9402440

[70] Ellis, P. et al. Reliable detection of somatic mutations in solid tissues by laser-capture microdissection and low-input DNA sequencing. *Nat Protoc.* **16**, 841–871 (2021). 33318691 10.1038/s41596-020-00437-6

[71] Moore, L. et al. The mutational landscape of human somatic and germline cells. *Nature* **597**, 381–386 (2021). 34433962 10.1038/s41586-021-03822-7

[72] Lee-Six, H. et al. The landscape of somatic mutation in normal colorectal epithelial cells. *Nature* **574**, 532–537 (2019). 31645730 10.1038/s41586-019-1672-7

[73] Cagan, A. et al. Somatic mutation rates scale with lifespan across mammals. *Nature* **604**, 517–524 (2022). 35418684 10.1038/s41586-022-04618-z PMC9021023

[74] Kirkwood, T. B. Evolution of ageing. *Nature* **270**, 301–304 (1977). 593350 10.1038/270301a0

[75] Milholland, B. et al. Differences between germline and somatic mutation rates in humans and mice. *Nat Commun.* **8**, 15183 (2017). 28485371 10.1038/ncomms15183 PMC5436103

[76] Cervantes, R. B., Stringer, J. R., Shao, C., Tischfield, J. A. & Stambrook, P. J. Embryonic stem cells and somatic cells differ in mutation frequency and type. *Proc Natl Acad Sci USA* **99**, 3586–3590 (2002). 11891338 10.1073/pnas.062527199 PMC122567

[77] Rouhani, F. J. et al. Mutational history of a human cell lineage from somatic to induced pluripotent stem cells. *PLoS Genet* **12**, e1005932 (2016). 27054363 10.1371/journal.pgen.1005932 PMC4824386

[78] Hart, R. & Setlow, R. Correlation between deoxyribonucleic acid excision-repair and life-span in a number of mammalian species. *Proc. Natl Acad. Sci. USA* **71**, 2169–2173 (1974). 4526202 10.1073/pnas.71.6.2169 PMC388412

[79] Britten, R. J. Rates of DNA sequence evolution differ between taxonomic groups. *Science* **231**, 1393–1398 (1986). 3082006 10.1126/science.3082006

[80] Goodman, M. Serological analysis of the systematics of recent hominoids. *Hum Biol* **35**, 377–436 (1963). 14063195

[81] Zhang, L. et al. Maintenance of genome sequence integrity in long-and short-lived rodent species. *Sci Adv* **7**, eabj3284 (2021). 34705500 10.1126/sciadv.abj3284 PMC8550225

[82] Larsen, E. C., Christiansen, O. B., Kolte, A. M. & Macklon, N. New insights into mechanisms behind miscarriage. *BMC Med.* **11**, 1–10 (2013). 23803387 10.1186/1741-7015-11-154 PMC3699442

[83] Vanneste, E. et al. Chromosome instability is common in human cleavage-stage embryos. *Nat Med.* **15**, 577 (2009). 19396175 10.1038/nm.1924

[84] Tollefsbol, T. O. & Gracy, R. W. Premature aging diseases: cellular and molecular changes. *BioScience* **33**, 634–639 (1983).

[85] Oshima, J., Sidorova, J. M. & Monnat, R. J. Jr Werner syndrome: clinical features, pathogenesis and potential therapeutic interventions. *Ageing Res Rev* **33**, 105–114 (2017). 26993153 10.1016/j.arr.2016.03.002 PMC5025328

[86] Furuichi, Y. Premature aging and predisposition to cancers caused by mutations in RecQ family helicases. *Ann N Y Acad Sci.* **928**, 121–131 (2001). 11795503 10.1111/j.1749-6632.2001.tb05642.x

[87] Fukuchi, K.-I, Martin, G. M. & Monnat, R. J. Jr Mutator phenotype of Werner syndrome is characterized by extensive deletions. *Proc. Natl Acad Sci USA* **86**, 5893–5897 (1989). 2762303 10.1073/pnas.86.15.5893 PMC297737

[88] Crabbe, L., Verdun, R. E., Haggblom, C. I. & Karlseder, J. Defective telomere lagging strand synthesis in cells lacking WRN helicase activity. *Science* **306**, 1951–1953 (2004). 15591207 10.1126/science.1103619

[89] Du, X. et al. Telomere shortening exposes functions for the mouse Werner and Bloom syndrome genes. *Mol Cell Biol.* **24**, 8437–8446 (2004). 15367665 10.1128/MCB.24.19.8437-8446.2004 PMC516757

[90] Salk, D., Au, K., Hoehn, H. & Martin, G. Cytogenetics of Werner’s syndrome cultured skin fibroblasts: variegated translocation mosaicism. *Cytogenet Genome Res* **30**, 92–107 (1981). 10.1159/000131596 7273860

[91] Melcher, R. et al. Spectral karyotyping of Werner syndrome fibroblast cultures. *Cytogenet Cell Genet* **91**, 180–185 (2000). 11173853 10.1159/000056841

[92] Martin, G. M., Hisama, F. M. & Oshima, J. Review of how genetic research on segmental progeroid syndromes has documented genomic instability as a hallmark of aging but let us now pursue Antigeroid syndromes! *J Gerontol A.* **76**, 253–259 (2021). 10.1093/gerona/glaa273 PMC7812512 33295962

[93] Rizza, E. R. et al. Xeroderma pigmentosum: a model for human premature aging. *J Investig Dermatol* **141**, 976–984 (2021). 33436302 10.1016/j.jid.2020.11.012 PMC7987754

[94] Shiloh, Y. & Lederman, H. M. Ataxia-telangiectasia (AT): an emerging dimension of premature ageing. *Ageing Res Rev* **33**, 76–88 (2017). 27181190 10.1016/j.arr.2016.05.002

[95] Dollé, M. E. et al. Increased genomic instability is not a prerequisite for shortened lifespan in DNA repair deficient mice. *Mutat Res.* **596**, 22–35 (2006). 16472827 10.1016/j.mrfmmm.2005.11.008

[96] Iourov, I. Y., Vorsanova, S. G., Liehr, T., Kolotii, A. D. & Yurov, Y. B. Increased chromosome instability dramatically disrupts neural genome integrity and mediates cerebellar degeneration in the ataxia-telangiectasia brain. *Hum Mol Genet* **18**, 2656–2669 (2009). 19414482 10.1093/hmg/ddp207

[97] Avancini C, et al. The auditory mismatch negativity reflects accelerated aging in adults with Down’s syndrome. *Alzheimers Dement.* **17**, e053537 (2021).

[98] Hasaart, K. A. et al. Mutation accumulation and developmental lineages in normal and Down syndrome human fetal haematopoiesis. *Sci Rep* **10**, 12991 (2020). 32737409 10.1038/s41598-020-69822-1 PMC7395765

[99] Panigrahi, R. G. et al. Hutchinson–Gilford progeria syndrome: a rare genetic disorder. *Case Rep Dentist* **2013**, 631378 (2013). 10.1155/2013/631378 PMC3830809 24288630

[100] De Sandre-Giovannoli, A. et al. Lamin a truncation in Hutchinson–Gilford progeria. *Science* **300**, 2055–2055 (2003). 12702809 10.1126/science.1084125

[101] McCord, R. P. et al. Correlated alterations in genome organization, histone methylation, and DNA–lamin A/C interactions in Hutchinson–Gilford progeria syndrome. *Genome Res* **23**, 260–269 (2013). 23152449 10.1101/gr.138032.112 PMC3561867

[102] Bikkul, M. U. et al. Telomere elongation through hTERT immortalization leads to chromosome repositioning in control cells and genomic instability in Hutchinson–Gilford progeria syndrome fibroblasts, expressing a novel SUN1 isoform. *Genes Chromosomes Cancer* **58**, 341–356 (2019). 30474255 10.1002/gcc.22711 PMC6590296

[103] Corso, C. et al. Molecular cytogenetic insights into the ageing syndrome Hutchinson–Gilford progeria (HGPS). *Cytogenet Genome Res* **111**, 27–33 (2005). 16093717 10.1159/000085666

[104] Liu, B. et al. Genomic instability in laminopathy-based premature aging. *Nat Med* **11**, 780–785 (2005). 15980864 10.1038/nm1266

[105] Niedernhofer, L. J. et al. A new progeroid syndrome reveals that genotoxic stress suppresses the somatotroph axis. *Nature* **444**, 1038–1043 (2006). 17183314 10.1038/nature05456

[106] De Boer, J. et al. Premature aging in mice deficient in DNA repair and transcription. *Science* **296**, 1276–1279 (2002). 11950998 10.1126/science.1070174

[107] Vogel, H., Lim, D.-S., Karsenty, G., Finegold, M. & Hasty, P. Deletion of Ku86 causes early onset of senescence in mice. *Proc Natl Acad Sci USA* **96**, 10770–10775 (1999). 10485901 10.1073/pnas.96.19.10770 PMC17958

[108] Hasty, P., Campisi, J., Hoeijmakers, J., Van Steeg, H. & Vijg, J. Aging and genome maintenance: lessons from the mouse? *Science* **299**, 1355–1359 (2003). 12610296 10.1126/science.1079161

[109] Mustjoki, S. & Young, N. S. Somatic mutations in “benign” disease. *N Engl J Med* **384**, 2039–2052 (2021). 34042390 10.1056/NEJMra2101920

[110] Park, S. et al. Clonal dynamics in early human embryogenesis inferred from somatic mutation. *Nature* **597**, 393–397 (2021). 34433967 10.1038/s41586-021-03786-8

[111] Erickson, R. P. Somatic gene mutation and human disease other than cancer. *Mutat Res* **543**, 125–136 (2003). 12644182 10.1016/s1383-5742(03)00010-3

[112] Koptides, M., Hadjimichael, C., Koupepidou, P., Pierides, A. & Deltas, C. C. Germinal and somatic mutations in the PKD2 gene of renal cysts in autosomal dominant polycystic kidney disease. *Hum Mol Genet* **8**, 509–513 (1999). 9949210 10.1093/hmg/8.3.509

[113] Bahar, R. et al. Increased cell-to-cell variation in gene expression in ageing mouse heart. *Nature* **441**, 1011–1014 (2006). 16791200 10.1038/nature04844

[114] Bartz, J., Jung, H., Wasiluk, K., Zhang, L. & Dong, X. Progress in discovering transcriptional noise in aging. *Int J Mol Sci* **24**, 3701 (2023). 36835113 10.3390/ijms24043701 PMC9966367

[115] Levy, O. et al. Age-related loss of gene-to-gene transcriptional coordination among single cells. *Nat Metabol* **2**, 1305–1315 (2020). 10.1038/s42255-020-00304-4 33139959

[116] Ren P, Zhang J, Vijg J. Somatic mutations in aging and disease. *GeroScience* **46**, 1–19 (2024). 10.1007/s11357-024-01113-3 PMC11336144 38488948

[117] Burnet FM. A modification of Jerne’s theory of antibody production using the concept of clonal selection. *Australian Journal of Science* (1957). 10.3322/canjclin.26.2.119 816431

[118] Tonegawa, S. Reiteration frequency of immunoglobulin light chain genes: further evidence for somatic generation of antibody diversity. *Proc Natl Acad Sci USA* **73**, 203–207 (1976). 813222 10.1073/pnas.73.1.203 PMC335869

[119] Zhu, M. et al. Somatic mutations increase hepatic clonal fitness and regeneration in chronic liver disease. *Cell* **177**, 608–621.e12 (2019). 30955891 10.1016/j.cell.2019.03.026 PMC6519461

[120] Jaiswal, S. et al. Age-related clonal hematopoiesis associated with adverse outcomes. *N Engl J Med.* **371**, 2488–2498 (2014). 25426837 10.1056/NEJMoa1408617 PMC4306669

[121] Goldman, E. A., Spellman, P. T. & Agarwal, A. Defining clonal hematopoiesis of indeterminate potential: evolutionary dynamics and detection under aging and inflammation. *Mol Case Stud* **9**, a006251 (2023). 10.1101/mcs.a006251 PMC10240836 36889927

[122] Bouzid, H. et al. Clonal hematopoiesis is associated with protection from Alzheimer’s disease. *Nat Med* **29**, 1662–1670 (2023). 37322115 10.1038/s41591-023-02397-2 PMC10353941

[123] Mahmoud, M. et al. Structural variant calling: the long and the short of it. *Genome Biol* **20**, 246 (2019). 31747936 10.1186/s13059-019-1828-7 PMC6868818

[124] Ahsan, M. U., Liu, Q., Perdomo, J. E., Fang, L. & Wang, K. A survey of algorithms for the detection of genomic structural variants from long-read sequencing data. *Nat Methods* **20**, 1143–1158 (2023). 37386186 10.1038/s41592-023-01932-w PMC11208083

[125] Yancik, R. & Ries, L. A. Aging and cancer in America: demographic and epidemiologic perspectives. *Hematol Oncol Clin North Am* **14**, 17–23 (2000). 10680069 10.1016/s0889-8588(05)70275-6

[126] Sadasivam, S. & DeCaprio, J. A. The DREAM complex: master coordinator of cell cycle-dependent gene expression. *Nat Rev Cancer* **13**, 585–595 (2013). 23842645 10.1038/nrc3556 PMC3986830

[127] Maslov, A. Y. & Vijg, J. Somatic mutation burden in relation to aging and functional life span: implications for cellular reprogramming and rejuvenation. *Curr Opin Genet Dev* **83**, 102132 (2023). 37931583 10.1016/j.gde.2023.102132 PMC10841402

[128] Bujarrabal-Dueso, A. et al. The DREAM complex functions as conserved master regulator of somatic DNA-repair capacities. *Nat Struct Mol Biol* **30**, 475–488 (2023). 36959262 10.1038/s41594-023-00942-8 PMC10113156

[129] Couteur, D. G. L. & Barzilai, N. New horizons in life extension, healthspan extension and exceptional longevity. *Age Ageing* **51**, afac156 (2022). 35932241 10.1093/ageing/afac156 PMC9356533

